# Returning to work at school during the COVID -19 pandemic, is it stressful for schoolteachers? Assessment of immediate psychological effects: a cross sectional study

**DOI:** 10.12688/f1000research.110720.2

**Published:** 2022-10-20

**Authors:** Sowmini Padmanabh Kamath, Prasanna Mithra, Jayashree K, Vaman Kulkarni, Jayateertha Joshi, Padmanabh Kamath, Bhaskaran Unnikrishnan, Keshava Pai

**Affiliations:** 1 Department of Pediatrics, Kasturba Medical College, Mangalore, Manipal Academy of Higher Education, Manipal, India; 2 Department of Community Medicine, Kasturba Medical College,Mangalore, Manipal Academy of Higher Education, Manipal, India; 3 Department of Community Medicine and Family Medicine., All India institute of Medical sciences (AIIMS), Bibinagar, Telengana, India; 4 Pediatric Surgery unit, Department of General Surgery, Kasturba Medical College, Mangalore, Manipal Academy of Higher Education, Manipal, India; 5 Department of Cardiology, Kasturba Medical College,Mangalore, Manipal Academy of Higher Education,Manipal, India; 6 Department of Psychiatry, Kasturba Medical College,Mangalore, Manipal Academy of Higher Education, Manipal, India

**Keywords:** Anxiety, counseling, COVID -19, depression, pandemics, schools, prevalence, school teachers

## Abstract

**Background:** The adoption of remote classes for students has been in vogue since the onset of the pandemic. Schools reopened in a phased manner after the second wave of coronavirus disease 2019 (COVID-19) in India. Reverting to the regular face-to-face teaching for students became a challenge to the teachers and students, especially at times when there was an impending third wave on the way. The study aimed to assess the presence of symptoms of depression, anxiety, and stress in teachers who attended reopened schools in the scenario of face-to-face classes. In addition, we studied the association of psychological symptoms with teachers' age groups, gender, school boards, and school institution type.

**Methods:** A cross-sectional study was conducted between October to December 2021 after schools had reopened. Data was collected using Google Form questionnaires in 124 schoolteachers. The Depression, Anxiety, and Stress Scale - 21 Items (DASS-21) questionnaire assessed the psychological symptoms.

**Results: **Of 124 schoolteachers, 108(87.1%) were female, 112 (90.3%) were from private institutions, and 70(56.5%) were from Central Board of Secondary Education (CBSE) school boards. The prevalence of depression, anxiety, and stress in teachers was 30.6%, 45.2%, and 20.2%, respectively. Nearly 80% of the female teachers expressed depression, anxiety, and stress symptoms. Amongst all the age groups, symptoms were higher in 40-49 group. We found anxiety to be statistically significant when compared with gender (p-0.042). We found no statistically significant differences concerning age groups, school boards, or school institutions with any psychological symptoms.

**Conclusions:** The prevalence of psychological symptoms was high among schoolteachers after schools reopened for regular face-to-face teaching. Gender was associated with anxiety in teachers. We agree that identifying teachers' symptoms and providing adequate psychological counseling/support would improve their mental health status and thereby the quality of teaching to students.

## Introduction

The coronavirus disease 2019 (COVID-19) pandemic has influenced significant economic, health, psychological, social, and educational changes across the globe ( World Health Organization (WHO)). With the onset of a pandemic, social distancing as a strategic measure was adopted worldwide to curtail the spread of COVID-19 infection. Schools and educational institutes had gone for closures and lockdowns in various countries ( Centers for Disease Control (CDC)). As per The United Nations Educational, Scientific and Cultural Organization (UNESCO), school closures as a social distancing measure varied in different countries ( UNESCO).

With school closures, the regular face-to-face teaching had to be stopped abruptly with a rapid shift to an online mode of education; this was unlike planned online learning. ^1^ Online learning used to prevail among higher classes and university students even during pre-COVID times. Moving to completely remote teaching across all age groups of children impacted the teachers, children, and their families as well ( EDUCASE). UNESCO reported stress and confusion among teachers in all parts of the world ( UNESCO).

In addition, teachers had to adjust rapidly to the remote teaching from regular face-to-face teaching to meet the expectations of students’ parents and school management. There was inadequate training for using digital resources and the insufficient provision of necessary equipment for online classes. ^2^ The instructional planning involved in transferring the teaching material into an online environment with maintaining its relevance was a challenge for the teachers ( The Brookings Institution). Along with the hectic professional life they led, they had increased family responsibilities during the pandemic. Earlier studies during the pandemic have shown that teachers have increased psychological symptoms with varying prevalence of anxiety, ^3^^–^^7^ stress, ^7^^,^^8^ and depression. ^3^^,^^7^^,^^9^

Another study showed that more than half of the teachers experience burnout; risk factors for burnout included skill development in distance teaching, increased workload on distance education, ^10^ and a new need for communication with parents because of increased involvement in remote teaching. ^11^ In addition, they experienced conflicts at work and within family, and there was lesser social support because of confinement. ^10^

Even before the pandemic, teachers have expressed that they experience psychological symptoms related to stress. ^12^^–^^14^ Teachers are always of prime importance since they are role models who influence students’ academic success and overall development. The teaching profession is otherwise also stressful, especially during pandemic times. Since the pandemic, the stress and anxiety levels have risen for reasons such as fear of getting infected, fear of the future, the health of oneself, their families, ^15^ economic crises, and uncertainties daily.

We presumed that going back to face-to-face teaching after reopening schools will reduce their psychological symptoms. However, teachers continued to have challenges when reverting because of the day-to-day uncertainties with the pandemic, ^16^ impending possibilities of upcoming waves, ^17^ children yet to receive vaccination ( Ministry of Health and Family Welfare), and extra responsibilities of teachers to maintain COVID appropriate behaviors among students ( Ministry of Education). A single study conducted in Spain on teachers found higher anxiety, stress, and depression levels with face-to-face teaching after school reopening. ^7^ However, there is minimal literature on anxiety, stress, and depression levels among Indian teachers returning to work at reopened schools in the time of the COVID-19 pandemic.

The Depression Anxiety Stress Scale questionnaire (DASS 21) has been widely used by numerous demographics ^18^^,^^19^ as well as numerous ethnic and cultural groupings ^20^^–^^22^ worldwide. The 21 item DASS questionnaire has been used in the Indian setting to evaluate psychological symptoms among distinct Indian communities. ^23^^,^^24^

The three-factor model of DASS 21 was found to be suitable for usage when the English version of the test was studied among Indian adults ^25^ and adolescents. ^26^ Confirmatory factor analysis of the Indian adult population's English-language DASS 21 evaluated general psychological discomfort. They inferred that there was no much evidence that the DASS-21 item responses correspond to the three latent subscales. ^25^

Teachers’ mental health monitoring becomes essential considering significant changes in teachers’ professional and personal lives. Therefore, the present study assessed the prevalence of anxiety, stress, and depression among teachers who attended reopened schools during the COVID-19 pandemic. Further, we studied associations of age, gender, type of school institutions, and school boards with anxiety, stress, and depression symptoms.

## Methods

### Study setting

A cross-sectional study was conducted among teachers at selected schools of Dakshina Kannada District, located in the coastal south Indian state of Karnataka. We collected the data by convenience sampling when the schools reopened following the second wave of COVID-19 infection (between October and December 2021).

### Study design

**Figure 1.**
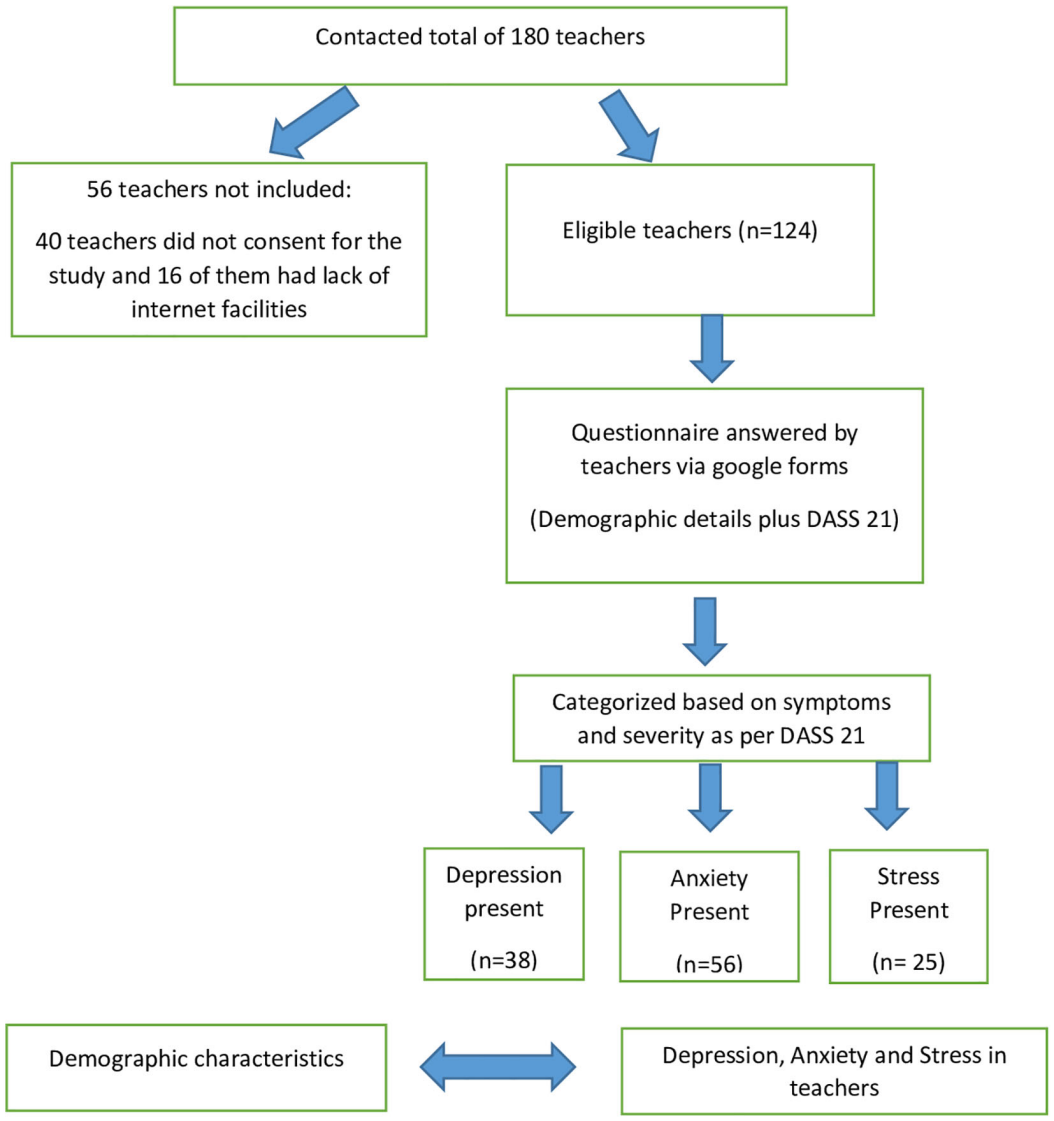
Study flow diagram.

This study follows ‘The Strengthening the Reporting of Observational Studies in Epidemiology (STROBE) statement guidelines. ^27^ A completed STROBE checklist can be found in the Reporting guidelines. ^28^ The study flow as per STROBE guidelines is depicted in the Figure 1.

### Sample size

We calculated a minimum sample size of 115, considering 80% power, 95% confidence level, relative precision of 10%, 20% non-response error, and an anticipated anxiety level in teachers to be 49.4%. ^7^

### Ethical considerations

The institutional ethical committee of Kasturba Medical College, Mangalore, approved the study (ethics committee approval number: IEC KMC MLR - 06/2020/184 dated 24/06/2020). We visited schools on a pre-informed date and took permission from the educational school authorities after explaining the purpose of the study. Teachers were provided the participant information sheet and informed consent was collected via an online platform (Consent form provided as *Extended data*). ^28^

### Inclusion and exclusion criteria

Teachers who were willing to participate in the study were included. Teachers who could not join the school because of their chronic medical conditions or lack of access to internet facilities were excluded.

### Data collection

We obtained the list of all the schools within our district from the office of the Block Education Officer (BEO). The sample size was divided equally among the public and private schools. In the ascending order of number of teachers, the schools were listed. Using a lottery technique, schools were then selected until we achieved the required sample size.

We obtained permission from the BEO and the respective school authorities, after explaining the details of the study. Data were collected using the online platform Google Forms to create the questionnaire as an infection control measure.

The link of the questionnaire with the participant information sheet containing details of study, the permission letter from the BEO and the IEC certificate were sent to the head teacher/principal of the selected school via WhatsApp and school email. The principal circulated the link in their respective schoolteachers’ WhatsApp group. A reminder message by the investigator with request to participate in the study was sent once after two weeks to the head teacher/principal. Informed consent was collected through the questionnaire link.

The questionnaire (provided as *Extended data*) ^28^ had two sections: section 1 for demographic data and section 2 included the questions from the standard questionnaire of Depression, Anxiety and Stress Scale - 21 Items (DASS-21 short version) questionnaires ( available from ePROVIDE). DASS-21 questionnaire was used to assess depression, anxiety, and stress levels. All the questions were compulsory. Participants could scroll down to the next question after answering the question in hand.

Being sociocultural acceptable and valid in Indian population, English version of DASS 21 was employed. The school teachers were well versed in the English language as well. Short version of DASS 21(English version) is a self-report questionnaire and is mapped out to ascertain the psychological states of anxiety, stress, and depression. The scale consists of 21 items with each statement having four response options (score 0-did did not apply to me at all; score 1-applied to me to some degree or some of the time; score 2-applied to me to a considerable degree or a good part of the time; and score 3-applied to me very much or most of the time). The responses specify how much the statement pertains to them over the last week. The questions are grouped for three scales, namely depression, anxiety, and stress, with seven items in each domain. The scores were summed up for pertinent items of each domain and multiplied by two to arrive at the final score. Based on the final scores for each domain, we classified the severity of symptoms as per cut-off scores into i) no symptoms, ii) mild, iii) moderate, iv) severe, and v) extremely severe symptoms.

### Operational definitions and study groups

Public schools include schools that are managed/aided by the Government. The Council for the Indian School Certificate Examinations (a private board of secondary education in India) conducts the Indian Certificate of Secondary Education (ICSE) examination. The Government of India manages the national level education board named “The Central Board of Secondary Education (CBSE)” for Indian public and private schools. The Karnataka state government operates the State Education Examination Board (State board). We grouped school boards as (CBSE+ICSE) versus state boards, and the type of schools was grouped as public versus private schools for comparison with anxiety, stress, and depression levels among teachers.

### Statistical analysis

IBM SPSS Statistics for Windows, Version 25.0. Armonk, NY: IBM Corp. was used to analyze the collected data. We expressed our results as proportions using appropriate tables. We analyzed the groups for their association with psychological symptoms by Chi-square test. To evaluate the link between the domains of stress, anxiety, and depression, we employed a partial correlation test and correlation coefficients were generated. A p-value of <0.05 was considered statistically significant.

## Results

A total of 180 teachers were contacted, of which 124 ^28^ responded to the questionnaire with a response rate of (68.89%). Of 124 teachers, 108 (87.1%) were female, 112 (90.3%) were from private institutions, and 70 (56.5%) were from CBSE school boards ( Table 1). The frequency and symptom severity of depression, anxiety, and stress as per the DASS 21 scale is depicted in Table 2.

**Table 1.** Basic demographic characteristics (CBSE: Central Board of Secondary Education).

Variables	n (%)
**Gender**	
Female	108(87.1)
Male	16(12.9)
**Age groups**	
20-29 years	6(4.8)
30-39 years	31(25)
40-49 years	59(47.6)
50-59 years	27(21.8)
60-65 years	1(0.8)
**Institution**	
Government aided institutes	12(9.7)
Private institution	112(90.3)
**School board**	
State board	54(43.5)
CBSE	70(56.5)

**Table 2.** Frequency  and symptom severity of depression, anxiety and stress levels in schoolteachers.

Psychological symptoms	n(%)
**Stress**	
None	99(79.8)
Mild	15(12.1)
Moderate	4(3.2)
Severe	4(3.2)
Extremely severe	2(1.6)
**Anxiety**	
None	68(54.8)
Mild	22(17.7)
Moderate	21(16.9)
Severe	4(3.2)
Extremely severe	9(7.3)
**Depression**	
None	86(69.4)
Mild	16(12.9)
Moderate	12(9.7)
Severe	7(5.6)
Extremely severe	3(2.4)

Of the sample, 45.2% of teachers indicated anxiety features, with 7.3% reporting features of extremely severe anxiety and 3.2% with severe anxiety. About 20.2% of teachers reported stress; 2.4% had extremely severe stress, and 5.6% had severe stress. Around 30.6% of teachers suffered from symptoms suggestive of depression, and extremely severe and severe symptoms were reported in 1.6%, and in 3.2% respectively. Symptoms of depression, anxiety and stress were seen in 78.95%, 80.36%, and 80% of female teachers, respectively. Similarly, symptoms of depression (52.63%), anxiety (50%), and stress (60%) were seen predominantly in teachers aged between 40-49 years.

Isolated depressive and stress symptoms were present in two (1.61%) and in eleven (8.87%) teachers respectively. About 34 instructors (27.42%) experienced both depressive and anxiety symptoms, whereas 23 teachers (18.55%) had both depressive and stress symptoms. Twenty-five teachers (20.16%) admitted having symptoms due to both stress and anxiety together. However, 23 (18.55%) teachers had all three psychological symptoms.

When stress was taken into account, depression and anxiety showed a weak association (coefficient: 0.285; p = 0.001). Depression and stress displayed a moderate correlation when anxiety was controlled for (coefficient is 0.559; p < 0.0001). Stress and anxiety showed a moderate link (coefficient is 0.504; p < 0.0001) while controlled for depression.

Furthermore, we grouped the symptoms as presence or absence of symptoms, namely as “No anxiety” and “anxiety present”; “No stress and stress present”; and “No depression and depression present” ( Table 3). We compared the gender, age groups, school boards, and school type with groups categorized with the presence or absence of symptoms for depression, anxiety, and stress. We found anxiety to be statistically significant when compared with gender (p-0.042). However, we found no statistically significant differences when we compared age groups, school boards, and school institutions with the psychological symptoms in teachers ( Table 4).

**Table 3.** Presence and absence of psychological symptoms in schoolteachers.

Symptomatology	Presence of symptoms N (%)	Absence of symptoms N (%)
Depression	38(30.6)	86(69.4)
Anxiety	56(45.2)	68(54.8)
Stress	25(20.2)	99(79.8)

**Table 4.** Comparison of demographic characteristics with depression, anxiety and stress levels among schoolteachers.

Characteristics		Depression No. (%)	p value	Anxiety No. (%)	p value	Stress No. (%)	p value
**Gender**	Male (n=16)	08(50.0)	0.072	11(68.8)	**0.042** ^*^	05(31.2)	0.236
	Female (n=108)	30(27.8)		45(41.7)		20(18.5)	
**Age groups (years)**	<40 (n=37)	11(29.73)	0.885	19(51.35)	0.366	6(16.22)	0.475
	≥40 (n=87)	27(31.03)		37(42.53)		19(21.84)	
**School board**	State (n=54)	15(27.8)	0.543	20(37.0)	0.110	12(22.2)	0.615
	CBSE (n=70)	23(32.9)		36(51.4)		13(18.6)	
**School institution**	Govt. aided (n=12)	01(08.3)	0.078	05(41.7)	0.798	01(08.3)	0.283
	Private (n=112)	37(33.0)		51(45.5)		24(21.4)	

* p value significant at < 0.05 level.

## Discussion

In our study, among the schoolteachers’ anxiety, stress, and depression symptoms were 45.2%, 20.2%, and 30.6%, respectively. These symptoms were in the context of face-to-face teaching after schools had reopened following the second wave of COVID-19 in India. Similar higher prevalence of anxiety (49.4%), stress (50.6%), and depression (32.2%) were present among teachers who had face-to-face teaching after schools reopened in Spain. ^7^

One significant change in the education system worldwide was adopting remote learning (e-learning) across all age groups. The higher stress levels during the COVID-19 pandemic could be because teachers had to adjust to the online teaching mode. ^29^ Home teaching had increased their workload, and there was a concomitant increase in anxiety, depression, and disturbances in sleep. With school closures, the teachers experienced stress and confusion because of the uncertainties regarding the duration of school closures and the unprecedented option of distance/online learning ( UNESCO).

A recent study found that some challenges and factors influence the use and receipt of online learning as a tool for higher education teaching. Among the challenges, the highest barriers were unstable or insufficient internet connectivity, lack of computers/laptops, and other technical issues. ^30^ Teachers’ perceptions on e-learning in India documented moderate and severe anxiety (each 5% by Hamilton rating scale for anxiety), with 37% of teachers reporting that the major drawback of online classes was a lack of face-to-face communication with students. ^5^ However, our study did not assess the teachers’ perceptions of e-learning.

A study that assessed the effect of online homeschooling on children, teachers, and 1-9 grades through the COVID-19 pandemic in China found that 17.6% of students (by parent-rated Strengths and Difficulties Questionnaire (SDQ)) had emotional or behavioral problems with more vulnerability in low-grade students. Elevated anxiety levels (by self-rating anxiety scale (SAS)) were seen in 17.2% of teachers and 9.6% of parents. ^6^ Their teacher’s anxiety levels were less than that reported in our study.

On similar grounds of returning to teaching at school in pandemic times, a survey assessed the differences in anxiety levels from the first day of joining to the end of one-month education. More than half (56.2%) of teachers did not show a change in the anxiety, 38.9% had decreased levels, and 4.9% of teachers had increased anxiety levels. The survey found communication within the school, the stress in teachers, and teaching instructions by virtual modes to be significant predictors of an increase in teachers’ anxiety. ^31^

Contrary to our study, university employees reported stress in 13% of participants, depression in 15.9%, anxiety in high to moderate levels in 13% (by DASS), and 43% reported high exhaustion at work. More than half of them (58.3%) had opined of having worsened overall well-being concerning COVID-19 work/life changes. ^9^

In the early part of the pandemic, an extensive survey conducted in China showed 58.1% of teachers being ‘very worried’ about the COVID-19 situation. ^32^ Our study had teachers’ anxiety levels more than three times that found in teachers in China (13.67% by the Generalized Anxiety Disorder tool (GAD-7)). ^4^ Gender, age, educational status, type of teachers, school location, worry, or fear level of teachers were associated with anxiety in a study done in China. ^4^ Unlike our findings, university teachers in Jordan, ^8^ reported severe distress in 31.4% of participants to mild to moderate distress in 38.2% (Kessler Distress scale-K10).

A systematic review and meta-analysis documented anxiety, depression, and stress in teachers as 17%, 19%, and 30%, respectively. ^33^ In yet another similar systematic review in teachers, the stress levels varied from 12.6% to 50.6%, anxiety levels between 10% and 49.4%, and depression levels between 15.9% and 28.9%. ^34^ In our study and a survey conducted among Spanish teachers, ^7^ the prevalence of stress, anxiety, and depression in teachers was higher. Daily uncertainties of the COVID situation, getting exposed to more contacts while commuting to school, and interacting with students were reasons for higher stress and anxiety. ^35^ In addition, the need to be more vigilant in maintaining the appropriate COVID behaviors for face-to-face classes with increased responsibilities to monitor students ( Ministry of Education; Learning Policy Institute), can affect their psychological symptoms to a large extent. Further, we had conducted the study when the schools had reopened after the second wave of COVID-19 infection, which had a devastating impact on Indians.

A study assessment of mental health status when the people returned to work in China noted that 10.8% had the criteria to fulfill post-traumatic stress disorder (PTSD), anxiety levels in 3.8%, stress in 1.5%, depression in 3.7%, and insomnia in 2.3%. ^36^ Their prevalence of psychological symptoms was lesser and contrasts our study and the Spanish study, ^7^ probably because of the faith induced by adopting preventive and social distancing measures before they started working. ^36^

A study in Poland found a negative relationship between social/marital/close links and coping of stress, anxiety, and depression among teachers; their psychological symptoms progressively worsened from the first to the second wave in Poland. ^37^ Another study assessed the teachers’ anxiety-related factors towards COVID-19 infection and education in school when they started having face-to-face classes; six reasons for infection-related and four factors for educational-related anxiety were found to be significant. Factors such as their anxiety regarding their safety/families (infection-related), anxiety about students’ home situations, and delay in students’ education (education-related) were strongly associated with anxiety. ^38^

The psychological symptoms were higher in schoolteachers ^3^^,^^5^^,^^6^ compared to university teachers ^4^^,^^8^^,^^9^ Our study mainly focused on schoolteachers. The higher prevalence among schoolteachers could be because more minor children need more supervision and monitoring of appropriate COVID behaviors than young adults in university.

It is a known fact that the most schoolteachers are female. Our study also had more participation from female teachers (87.1%) with higher levels of symptomatology in them. Similarly, earlier studies have shown female teachers to have higher psychological symptoms through COVID-19 pandemic, ^33^^,^^34^ and this is true even in the pre-COVID times. ^12^^,^^13^ The present study showed a significant association between differences in gender with anxiety in teachers; such similar results were seen in previous studies. ^4^^,^^7^^,^^39^

The new mode of online teaching to conduct classes with a heavy workload at school, homeschooling and attending family duties, extra responsibilities of parenting, child-care, and multitasking to meet the family demands ^40^ may be reasons for the female teachers to be more symptomatic. Professional and personal commitments increase stress levels. Our study did not assess these factors separately.

The present study showed psychological symptoms to be predominant in the teachers between 40-49 years. Similar findings of older teachers (>47 years) having more significant anxiety and younger teachers having higher stress scores were seen among Spanish teachers. ^13^ However, age groups did not show a significant association with any of the symptoms in our study. With age, there is probably a building of resilience with frequent exposures to various stressors over time, molding them to have stabler emotional management and lower psychological symptoms. ^41^

In the present study, teachers working in CBSE+ICSE school boards and private institutions had higher symptoms than state boards and Government aided schools, probably because of more response rates from the former. There was no significant association of school boards and type of school institution (Government aided versus private) with psychological symptoms with limited literature available until date. The study’s limitations include a small sample size and lesser participation of teachers from public/government-aided institutions. In addition, results may not reflect the teachers’ opinions from other areas of the state/country. The causality of psychological symptoms was not established and conjecture that it was a combination of reopening after a hiatus, challenges of practicing and enforcing new behaviors’ and fear of unpredictable resurgence of infection. Furthermore, the symptoms of stress, anxiety, and depression can influence one another. Teachers' stress, depression, and anxiety may be significantly influenced by COVID-related socioeconomic and health challenges, however this was not explored. The study’s strengths were that we used a standard questionnaire to assess the prevalence of symptoms of anxiety, stress, and depression (DASS 21) in teachers. We compared the symptom prevalence with school boards and public versus private school institutions, which has not been assessed in previous studies.

## Conclusion

After schools reopened, teachers had a higher prevalence of psychological symptoms when reverting to face-to-face classes. Gender was significantly associated with anxiety. Teachers’ role in shaping children stands beyond the mundane syllabus, courses, and exams. They are considered torchbearers of hope for a bright future for young children, and if they are affected by psychological symptoms, it will influence the quality of teaching. Thus, it is essential to identify the psychological symptoms teachers face during the COVID-19 pandemic crisis and encourage them to seek the professional help of counselors to adapt smoothly during current so-called new-normal situations. Further creating an encouraging/positive working atmosphere that will help teachers get connected and aid them in sharing matters that concern them would help them.

## Data availability

### Underlying data

Open Scientific Framework: Returning to work at school during COVID -19 pandemic, is it stressful for schoolteachers: Assessment of immediate psychological effects. https://doi.org/10.17605/OSF.IO/8VHJ3 ^33^

This dataset contains the following underlying data:

• Excel data F1000 research.xlsx
• Data Key.docx

### Extended data

Open Scientific Framework: Returning to work at school during COVID -19 pandemic, is it stressful for schoolteachers: Assessment of immediate psychological effects. https://doi.org/10.17605/OSF.IO/8VHJ3 ^33^

This dataset contains the following underlying extended data:

• DASS-21 PDF.pdf
• Participant Information Sheet and Informed Consent-F1000 research.docx
• Questionnaire-F1000 research.docx
• Study-flow-figure-F1000-research.jpg

Data are available under the terms of the Creative Commons Attribution 4.0 International license (CC-BY 4.0).

## Reporting guidelines

Open Scientific Framework: STROBE checklist for “Returning to work at school during COVID -19 pandemic, is it stressful for schoolteachers: Assessment of immediate psychological effects.” https://doi.org/10.17605/OSF.IO/8VHJ3 ^33^
