# Supplementary material for: Returning to work at school during the COVID -19 pandemic, is it stressful for schoolteachers? Assessment of immediate psychological effects: a cross sectional study
Source: F1000Res. 2022 Oct 20;11:751. Originally published 2022 Jul 6. [Version 2] doi: 10.12688/f1000research.110720.2 (PMC9617070; doi:10.12688/f1000research.110720.2)
Supplement: Peer review history

# Peer review: Reviewer response for version 2

**DOI:** 10.5256/f1000research.139930.r153786

**Published:** 2022-10-28

**Authors:** Ajay Risal

**Affiliations:** 1 Department of Psychiatry, Kathmandu University School of Medical Sciences, Dhulikhel Hospital, Kathmandu University Hospital, Dhulikhel, Nepal

Since the authors have revised the manuscript as per the reviewer's comments, I approve the manuscript to be indexed.

Is the work clearly and accurately presented and does it cite the current literature?

Yes

If applicable, is the statistical analysis and its interpretation appropriate?

Partly

Are all the source data underlying the results available to ensure full reproducibility?

Yes

Is the study design appropriate and is the work technically sound?

Yes

Are the conclusions drawn adequately supported by the results?

Yes

Are sufficient details of methods and analysis provided to allow replication by others?

Yes

Reviewer Expertise:

MD Psychiatry, Ph.D. Clinical Medicine

I confirm that I have read this submission and believe that I have an appropriate level of expertise to confirm that it is of an acceptable scientific standard.

# Peer review: Reviewer response for version 2

**DOI:** 10.5256/f1000research.139930.r153785

**Published:** 2022-10-28

**Authors:** Rajesh Mithur

**Affiliations:** 1 Department of Psychiatry, Yenepoya Medical College, Yenepoya Deemed to Be University, Mangalore, Karnataka, India

Dear Authors, 

 I'm happy to read your version 2 of the article with suitable modifications. With the above modifications, I believe the article is suitable for indexing.

 Thank You,

 Dr. Rajesh M, MD,(Ph.D.)

Is the work clearly and accurately presented and does it cite the current literature?

Yes

If applicable, is the statistical analysis and its interpretation appropriate?

Partly

Are all the source data underlying the results available to ensure full reproducibility?

Yes

Is the study design appropriate and is the work technically sound?

Yes

Are the conclusions drawn adequately supported by the results?

Partly

Are sufficient details of methods and analysis provided to allow replication by others?

Yes

Reviewer Expertise:

Neuropsychiatry, Adult psychiatry

I confirm that I have read this submission and believe that I have an appropriate level of expertise to confirm that it is of an acceptable scientific standard.

# Peer review: Reviewer response for version 1

**DOI:** 10.5256/f1000research.122353.r148222

**Published:** 2022-09-05

**Authors:** Rajesh Mithur

**Affiliations:** 1 Department of Psychiatry, Yenepoya Medical College, Yenepoya Deemed to Be University, Mangalore, Karnataka, India

A good study indeed done in COVID times. Kindly provide the following clarifications and address the corrections:

1. "India today" reference may not be appropriate for a scientific journal.
2. "Returning to work at school during the COVID -19 pandemic, Is it stressful for school teachers?" - the title gives an impression that the teachers faced stress due to schools reopening after the COVID pandemic. Kindly specify in the study that the stress teachers are facing is due to the COVID-19 pandemic and not due to school reopening per se. The causality of the stress was not established in the study. The psychological symptoms are seen in these teachers mostly due to the COVID pandemic.
3. The overview of the DASS 21 scale quotes, “The DASS is based on a *dimensional* rather than a *categorical* conception of psychological disorder. The assumption on which the DASS development was based is that the differences between the depression, anxiety, and stress experienced by normal subjects and the clinically disturbed, are essential differences in degree. The DASS, therefore, has no direct implications for the allocation of patients to discrete diagnostic categories postulated in classificatory systems such as the DSM and ICD.” ^1^ - The overview quotes that depression, anxiety, and stress are taken as a single dimension in the scale and not as separate unique entities, hence, percentage depictions of individual entities have to be clarified. There will be a significant overlap of stress, depression, and anxiety among patients which has to be specified.
4. Page 9: **casualty** term has to be corrected. **Causality** is not established is a main drawback of the study.
5. Limitation: COVID-related socioeconomic issues themselves may be a significant contributor to stress and cause of depression and anxiety which was not investigated.
6. References: 6, 7, 18, 22, 23, 24, 25, 27, 28, 31 has to be in proper reference style.

Is the work clearly and accurately presented and does it cite the current literature?

Yes

If applicable, is the statistical analysis and its interpretation appropriate?

Partly

Are all the source data underlying the results available to ensure full reproducibility?

Yes

Is the study design appropriate and is the work technically sound?

Yes

Are the conclusions drawn adequately supported by the results?

Partly

Are sufficient details of methods and analysis provided to allow replication by others?

Yes

Reviewer Expertise:

Neuropsychiatry, Adult psychiatry

I confirm that I have read this submission and believe that I have an appropriate level of expertise to confirm that it is of an acceptable scientific standard, however I have significant reservations, as outlined above.

## References

[1] : Confirmatory Factor Analysis of Three Versions of the Depression Anxiety Stress Scale (DASS-42, DASS-21, and DASS-12) in Polish Adults. *Front Psychiatry* .2021;12: 10.3389/fpsyt.2021.770532 770532 10.3389/fpsyt.2021.770532 35058818PMC8764392

# Response

**Published:** 2022-10-16

**Authors:** Sowmini Padmanabh Kamath

**Affiliations:** Kasturba Medical College, Mangalore, India

1. "India today" reference may not be appropriate for a scientific journal.

**Thank you for the suggestion. We have changed the reference as advised (in the sixth paragraph of the introduction).**

 2. "Returning to work at school during the COVID -19 pandemic, Is it stressful for school teachers?" - the title gives an impression that the teachers faced stress due to schools reopening after the COVID pandemic. Kindly specify in the study that the stress teachers are facing is due to the COVID-19 pandemic and not due to school reopening per se. The causality of the stress was not established in the study. The psychological symptoms are seen in these teachers mostly due to the COVID pandemic

**Thank you. We have described this in the limitation section of the discussion.** **We agree that the causality of stress was not established and conjecture that it was a combination of reopening after a hiatus, challenges of practicing and enforcing new behaviors and fear of unpredictable resurgence of infection.**

 3.The overview of the DASS 21 scale quotes, “The DASS is based on a dimensional rather than a categorical conception of psychological disorder. The assumption on which the DASS development was based is that the differences between the depression, anxiety, and stress experienced by normal subjects and the clinically disturbed, are essential differences in degree. The DASS, therefore, has no direct implications for the allocation of patients to discrete diagnostic categories postulated in classificatory systems such as the DSM and ICD.”1 - The overview quotes that depression, anxiety, and stress are taken as a single dimension in the scale and not as separate unique entities, hence, percentage depictions of individual entities have to be clarified. There will be a significant overlap of stress, depression, and anxiety among patients, which has to be specified **.**

**Thank you for the suggestion.** **Depictions of individual entities and overlap of psychological symptoms have been included in the results section (third paragraph).**

 4. Page 9: casualty term has to be corrected. Causality is not established is a main drawback of the study.

**Thank you for the suggestion. The spelling is corrected and changes made are in the limitation section of the discussion.**

 5. Limitation: COVID-related socioeconomic issues themselves may be a significant contributor to stress and cause of depression and anxiety, which was not investigated.

**Thank you for the suggestion. I have added this point in the limitation section of the discussion.**

****

 6. References: 6, 7, 18, 22, 23, 24, 25, 27, 28, 31 has to be in proper reference style.

**Thank you. Vancouver reference style has been adopted.**

# Peer review: Reviewer response for version 1

**DOI:** 10.5256/f1000research.122353.r145634

**Published:** 2022-08-05

**Authors:** Ajay Risal

**Affiliations:** 1 Department of Psychiatry, Kathmandu University School of Medical Sciences, Dhulikhel Hospital, Kathmandu University Hospital, Dhulikhel, Nepal

It is a very good study that assessed the psychological symptoms, mainly anxiety, depression, and stress, among the school teachers of Karnataka State in India while the schools became open for physical classes in the post-COVID phase.

 It has used a valid instrument DASS for assessing the psychology of the teachers. However, the following two concerns need clarification and explanation before this research can be considered acceptable for indexing:

1. Which version of DASS-21 was used for the assessment? It is not clear. Is it acceptable to use the English version (if it was the case) for all the participants whose mother tongue is supposedly not English for assessing their psychological issues? Will this procedure be socio-culturally accepted or valid? Is this methodology culture-sensitive? I think this concern needs good clarification and explanation.
2. DASS-21 was able to assess the individual depression (D), anxiety (A), and stress (S) symptoms independently as per the results you have shown, but it is not clear how many participants had combined D+A or D+S, A+D, or D+A+S symptoms. Was this result not important? Does not D symptoms affect A or S symptoms or vice versa? How were those effects analyzed? Were not D symptoms confounder for A or S symptoms; or A confounder to D and S; or S confounder to D or A? How were these possible confounding issues handled? I think this should also be the finding. These issues must get space in the Methodology and Results or at least in the Discussion as a possible limitation.

Otherwise, it is a good study.

Is the work clearly and accurately presented and does it cite the current literature?

Yes

If applicable, is the statistical analysis and its interpretation appropriate?

Partly

Are all the source data underlying the results available to ensure full reproducibility?

Yes

Is the study design appropriate and is the work technically sound?

Yes

Are the conclusions drawn adequately supported by the results?

Yes

Are sufficient details of methods and analysis provided to allow replication by others?

Yes

Reviewer Expertise:

MD Psychiatry, Ph.D. Clinical Medicine

I confirm that I have read this submission and believe that I have an appropriate level of expertise to confirm that it is of an acceptable scientific standard, however I have significant reservations, as outlined above.

# Response

**Published:** 2022-10-16

**Authors:** Sowmini Padmanabh Kamath

**Affiliations:** Kasturba Medical College, Mangalore, India

1. Which version of DASS-21 was used for the assessment? It is not clear. Is it acceptable to use the English version (if it was the case) for all the participants whose mother tongue is supposedly not English for assessing their psychological issues? Will this procedure be socio-culturally accepted or valid? Is this methodology culture-sensitive? I think this concern needs good clarification and explanation.

**Thank you for the suggestion. We have addressed the query with an explanation in the introduction section (para 7 and 8) and in the methodology section (DASS 21 questionnaire).**

 2. DASS-21 was able to assess the individual depression (D), anxiety (A), and stress (S) symptoms independently as per the results you have shown, but it is not clear how many participants had combined D+A or D+S, A+D, or D+A+S symptoms. Was this result not important? Does not D symptoms affect A or S symptoms or vice versa? How were those effects analyzed? Were not D symptoms confounder for A or S symptoms; or A confounder to D and S; or S confounder to D or A? How were these possible confounding issues handled? I think this should also be the finding. These issues must get space in the Methodology and Results or at least in the Discussion as a possible limitation.

**Thank you for the suggestion. We have now included the number of school teachers with combined psychological symptoms in the results section (para 3).**

**Yes, the symptoms affect each other to some extent and vice versa. The symptoms can be a confounder with each other. To evaluate the link between the domains of stress, anxiety, and depression, we employed a partial correlation test and correlation coefficients were generated.**

**T** **hese issues have been incorporated in the statistical analysis section of the methodology and in the results section (para 4) with correlation coefficients. In the discussion section, it has been included as a limitation.**
